# The Role of Extracellular Carbonic Anhydrase in Biogeochemical Cycling: Recent Advances and Climate Change Responses

**DOI:** 10.3390/ijms22147413

**Published:** 2021-07-10

**Authors:** Nur Ili Hamizah Mustaffa, Mohd Talib Latif, Oliver Wurl

**Affiliations:** 1 Department of Earth Science and Environment, Faculty of Science and Technology, Universiti Kebangsaan Malaysia, Bangi 46300, Selangor, Malaysia; talib@ukm.edu.my; 2 Centre for Marine Sensors, Institute for Chemistry and Biology for the Marine Environment, Carl von Ossietzky University of Oldenburg, 26382 Wilhelmshaven, Germany

**Keywords:** sea surface microlayer, diatom, carbon-concentrating mechanism, ocean acidification

## Abstract

Climate change has been predicted to influence the marine phytoplankton community and its carbon acquisition strategy. Extracellular carbonic anhydrase (eCA) is a zinc metalloenzyme that catalyses the relatively slow interconversion between HCO_3_^−^ and CO_2_. Early results indicated that sub-nanomolar levels of eCA at the sea surface were sufficient to enhance the oceanic uptake rate of CO_2_ on a global scale by 15%, an addition of 0.37 Pg C year^−1^. Despite its central role in the marine carbon cycle, only in recent years have new analytical techniques allowed the first quantifications of eCA and its activity in the oceans. This opens up new research areas in the field of marine biogeochemistry and climate change. Light and suitable pH conditions, as well as growth stage, are crucial factors in eCA expression. Previous studies showed that phytoplankton eCA activity and concentrations are affected by environmental stressors such as ocean acidification and UV radiation as well as changing light conditions. For this reason, eCA is suggested as a biochemical indicator in biomonitoring programmes and could be used for future response prediction studies in changing oceans. This review aims to identify the current knowledge and gaps where new research efforts should be focused to better determine the potential feedback of phytoplankton via eCA in the marine carbon cycle in changing oceans.

## 1. Introduction

Extracellular carbonic anhydrase (eCA) is a zinc metalloenzyme that accelerates the slow interconversion between bicarbonate ions (HCO_3_^−^) and carbon dioxide (CO_2_) to the equilibrium concentration at the cell surface [1]. eCA has been widely found in mammals [2], plants and phytoplankton [3], and prokaryotes [4]. In general, there are seven CA gene classes that have been recognized in photosynthetic organisms, identified as α-, β-, ϒ-, δ-, ζ-, θ- [3,5] as well as a recently discovered ι-CA gene class [6]. Meanwhile, the η-CA gene class has been found within the malaria pathogen *Plasmodium* sp. [7]. The first five gene classes (α, β, ϒ, θ and η) are different in terms of their primary structure [8] but share a common feature of bound zinc (Zn^2+^) on their activation site [9]. The δ-CA (TWCA1) [10,11] and ζ-CA (CDCA) [12] classes with the capability to bind with alternative metal cofactors as well as Zn^2+^, such as cobalt (Co^2+^) and cadmium (Cd^2+^), respectively, have been identified in the diatom *Thalassiosira weisflogii* (*T. weisflogii*). The δ- and ζ- classes are likely to be the major CA classes that facilitate CO_2_ supply in centric diatoms [13] as a carbon-concentrating mechanism (CCM). More recently, Jensen, et al. [6] discovered a new ι-CA class in *Thalassiosira pseudonona (T. pseudonona),* which unusually prefers manganese (Mn^2+^) to Zn^2+^ as a cofactor. Overall, the gene distributions of CA in microalgae cells vary between species even if they belong to the same family [14]. For additional information on the function, physiological relevance, and diverse CA expression in microalgae, we refer to a recent review [14].

eCA expression is highly responsive to environmental changes, particularly at low aqueous CO_2_ concentrations [15]. Previous studies have shown that the levels of eCA expression differ significantly between phytoplankton species based on laboratory experiments [16,17,18]. The availability of inorganic carbon, light levels and pH [19,20], as well as the phytoplankton growth stage [18], are important factors in the regulation of eCA activity. The taxonomic composition and cell size of a phytoplankton community also influence the level of eCA expression [21,22]. The different levels of eCA among species provide evidence that the mechanism of inorganic carbon (C_i_) acquisition in phytoplankton is species-dependent and eCA is produced when demand for CO_2_ exceeds the rate of uncatalyzed HCO_3_^−^ to CO_2_ conversion [16,17,23].

Under a future of climate change, marine photoautotrophs will undergo complex changes in their physiology, driven by increasing sea-surface temperatures, continuing ocean acidification, and changing light conditions [24,25]. Numerous laboratory studies have already described the effect of ocean acidification on C_i_ acquisition of microalgae, specifically diatom and their eCA activity [26,27,28]. Thus, it is not surprising that these changes include the expression of eCA in phytoplankton, particularly those residing in the near-surface layer. In this review, we focus on the available studies on eCA in the marine environment, including biological function and current approaches to understanding the eCA in changing oceans.

## 2. Biological Function of eCA in the Marine Environment

For decades, CA has been known to exist in many photosynthetic organisms and to be involved in CCMs, which help the cell to produce biomass via photosynthesis, particularly in a CO_2_-limited environment [3]. At the alkaline pH of seawater (pH 7.8–8.4), C_i_ predominantly exists in ionic forms, whereby approximately 90% is present as HCO_3_^−^, 9% as carbonate ions (CO_3_^2−^), and 1% present as CO_2_, the substrate for the CO_2_-fixing enzyme ribulose-1,5-bisphosphate carboxylase/oxygenase (RubisCO) [29,30]. RubisCO has a lower affinity for CO_2_ and, at the relatively low CO_2_ concentration found in the marine environment, the activity of this enzyme is less than half-saturated [3,31]. It was reported that the CO_2_ concentration at an air-equilibrated water surface is lower (13 µM at 20 °C) than typical values of the half-saturation constant (K_C_) of RubisCO in diatoms (K_C_ = 23–68 µM) [32], cyanobacteria (K_C_ = 100–180 µM) [33], and haptophytes (K_C_ = 15–24 µM) [34]. To overcome the CO_2_ limitation and slow diffusion rate in seawater, photosynthetic organisms evolved CCMs to increase the concentration of CO_2_ in the vicinity of the cell internal RubisCO site [31]. These mechanisms include active uptake of both extracellular HCO_3_^−^ and CO_2_ as carbon sources for photosynthesis. RubisCO-mediated carboxylation competes with the oxygenation of ribulose 1,5-biphosphate (RuBP), which reduces carbon fixation and promotes photorespiration [35]. However, the degree to which these two competitive reactions occur depends on the (O_2_) and CO_2_ concentrations at the active site of RubisCO and the relative affinity of the enzyme to these gases.

**Figure 1:**
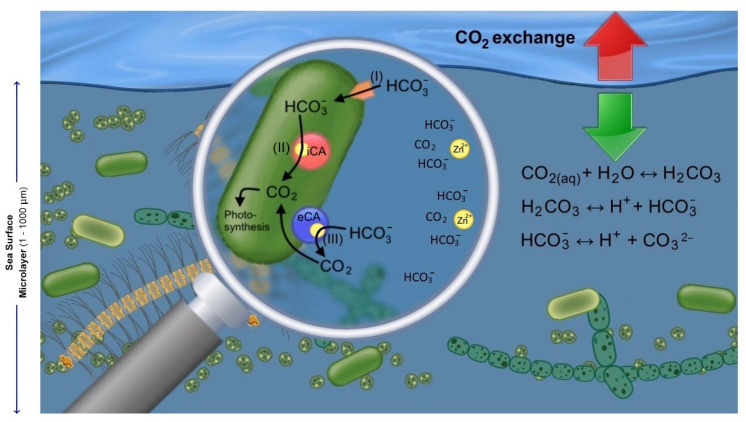
The role of extracellular (eCA) and internal carbonic anhydrase (iCA) in the sea surface microlayer (SML). Equations are given for the hydration of CO_2_. (i) Alternative carbon acquisition through direct uptake of HCO_3_^−^. (ii) Internal conversion of HCO_3_^−^ to CO_2_ by iCA. (iii) Catalytic conversion of HCO_3_^−^ to CO_2_ by eCA within the cell’s diffusive layer, and CO_2_ diffusion through the cell’s membrane.

In phytoplankton and aquatic macrophytes, CA can be located either in periplasmic space (eCA) or attached to the outer cell wall and/or in the chloroplast (internal CA, iCA) (Figure 1) [36,37,38]. There are lines of evidence supporting the role of eCA in some microalgae CCM, particularly diatoms, and eCA expression is induced under low CO_2_ concentration [23,30,39]. CCM consists of a C_i_ pump, CA enzyme to equilibrate HCO_3_^−^ to CO_2_, and a compartment of RubisCO such as pyrenoid or carboxysome [33]. The function of eCA in CCMs is mainly to convert available HCO_3_^−^ to CO_2_ close to the cell membrane and facilitate CO_2_ transport through the cell’s membrane by diffusion [38]. At low partial pressures of CO_2_ (pCO_2_) in the surrounding medium, i.e., seawater, the thin diffusion layer around the cell becomes depleted rather quickly compared with the larger bulk phase outside of the diffusion layer. Thus, eCA accelerates the slow dehydration of HCO_3_^−^ to CO_2_ within the boundary layer, increasing the surface CO_2_ concentration for fixation by Rubisco [3]. Besides, eCA also functions to recover leaked CO_2_ from the cell and convert it to HCO_3_^−^ [40], implying that the presence or absence of eCA allows more energy-efficient C_i_ recycling in CO_2_ and HCO_3_^−^ users [41].

The role of eCA in marine biogeochemical cycling is highlighted by the fact that eCA is ubiquitous and requires the binding of trace elements on its activation site, such as Zn, Cd, and Co [42,43], and, more recently discovered, Mn [6]. In 1994, Morel and co-workers proposed the “zinc hypothesis” where the low levels of Zn in surface water may limit CO_2_ uptake and the growth rate of *T. weissflogii* through eCA. Based on this finding, the low level of Zn in seawater has been suggested to reflect the distribution of eCA in seawater, which was proposed to be at nanomolar levels [44], but only recently confirmed with the development of an analytical technique to quantify eCA in seawater [18]. Analysis with the same diatom, *T. weissflogii,* showed that Cd [12,45] and Co [11] could partially replace Zn in CA by 50%, depending on the species, when the metals were present at concentrations typical of surface seawater. However, further analyses with chlorophytes and prymnesiophytes [46] indicated that Cd only acted as a nutrient in a narrow species-specific concentration range. For this reason, the replacement of Zn with Cd or Co has been suggested to be species-specific [47]. An activation of CA by Mn was proposed more recently as a ubiquitous sub-class of CA [6], and eCA could potentially be important in the understanding of Mn distribution in the oceans. A recent field study by Morel, et al. [48] in the eastern tropical South Pacific revealed that the substitution of Cd and Co for Zn occurred when dissolved Zn levels were extremely low and not necessarily with the lowest pCO_2_ conditions. This further suggests that diatoms in the marine environment may be co-limited by Zn-Cd-Co and CO_2_ [49]. To date, the cellular quotas of Zn attached to CA in marine phytoplankton remain an open question. Subhas, et al. [50] estimated the use of Zn quota by marine phytoplankton assemblages from the North Pacific Ocean to be in the range of 10–40% using Zn/phosphate and CA/particulate organic carbon (CA/POC) ratios. The estimated values are 10 times lower than our previous estimation from a laboratory experiment using monoculture solutions [18]. Some species are likely to utilize Cd, Co or even newly found Mn as a cofactor, and such estimations are likely to be very uncertain within natural assemblages. Trace metal quotas in marine phytoplankton also depend on cell size [49,51]. A holistic approach is needed to resolve the coupling of marine trace metal chemistry to total CA expression and activity in natural phytoplankton assemblages.

## 3. Extracellular Carbonic Anhydrase in a Changing Ocean

Under future climate change, marine photoautotrophs will undergo complex changes in their physiology, driven by increasing sea-surface temperatures, continuing ocean acidification, and changing light conditions [24]. As outlined above, changes could include the expression of eCA and changes within CCMs of phytoplankton communities. Between 1994 and 2007, it was reported that the amount of oceanic carbon increased by 34 ± 4 Pg C, which represents over 31 ± 4% of anthropogenic CO_2_ emissions [52]. Future concentrations of CO_2_ in the atmosphere are projected to reach ∼1000 µatm by 2100 if anthropogenic emissions are ongoing at the current rate [53] and thus will be taken up by the ocean through the sea surface microlayer (SML). The increased CO_2_ uptake by the ocean will influence the seawater chemistry, increase acidity, and shift the dissolved inorganic carbon system from carbonate (CO_3_^2−^) towards HCO_3_^−^ and CO_2_ [54,55]. This phenomenon is termed ocean acidification [54]. The air-sea CO_2_ exchange depends not only on temperature, salinity, and physical mixing of water, but also on the photosynthesis and respiration of plankton communities to maintain an air–sea CO_2_ gradient as a driving force for the exchange. Organisms in the euphotic zone will be exposed to a higher CO_2_ environment with the consequence of lower pH. Consequently, their physiologies will respond to these changes in marine carbonate chemistry. Ocean acidification generally affects the species composition of phytoplankton assemblages [56], changes the cellular mechanisms involved in the acquisition of inorganic carbon, and negatively affects the physiology of calcifying organisms such as coccolithophores [57]. Laufkötter, et al. [58] estimated a decrease in global average phytoplankton net primary production of 6.5% within 50 years of observation (1960–2006) due to changes in climate-relevant factors, with a consequence of reduced efficiency of the biological pump and thus the ocean’s capability to capture anthropogenic CO_2_ in the deep ocean.

Many laboratory studies have already described the effect of ocean acidification on the C_i_ acquisition of diatoms and their eCA activity (Table 1) [26,27,28]. In most cases, increased CO_2_ levels inhibit the eCA activity of diatoms. Hence, indirect uptake of HCO_3_^−^ via the eCA pathway is likely to be reduced under future elevated CO_2_ levels in the oceans. Nevertheless, diatoms display a high diversity in terms of C_i_ acquisition strategies, which can take both HCO_3_^−^ and CO_2_ [16,59]. *T. weissflogii*, where HCO_3_^−^ is the main C_i_ species taken up during low pCO_2_, showed the highest eCA expression under low pCO_2_ (36 µatm, pH = 9.1) [26] and decreased eCA expression by more than 50% after exposure to moderate pCO_2_ levels (180 µatm and 360 µatm). The eCA expression was close to the detection limit under high pCO_2_ (1800 µatm). Overall, under C_i_ limitation, eCA becomes an essential pathway for photosynthetic carbon fixation in *T. weissflogii*. Decreased eCA expression of *T. weissflogii* under high pCO_2_ has been observed in other studies [27,28]. *T. weissflogii* exhibited significantly higher photosynthetic oxygen evolution rates at low CO_2_ or HCO_3_^−^ levels, suggesting that *T. weissflogii* has higher affinities for CO_2_ or HCO_3_^−^ when their concentrations are not sufficient to support saturated growth and photosynthesis [28]. Gao and Campbell [25] suggested that CCMs in diatoms potentially link to multiple metabolic pathways that differ between species. For instance, under non normal conditions, *T. weissflogii* employs C_4_ pathways as additional CCMs before RubisCO-aided carboxylation [60], and the δ-CA of *T. weissflogii* can catalyse the hydration of CO_2_ and increase the HCO_3_^−^ concentration intracellularly [61]. *Phaeodactylum tricornutum* (*P. tricornutum*), however, relies solely on biophysical CCMs in which HCO_3_^−^ is pumped into the cell and converted into CO_2_ by the eCA in the chloroplast [27,28]. This explains the low eCA expression with increasing CO_2_ concentrations in *P. tricornutum* [26]. Trimborn, et al. [40] observed that the eCA activities of *T. pseudonana* were not affected by CO_2_ levels, suggesting that eCA plays a negligible role in its carbon acquisition strategy, but eCA plays an important role in bloom-forming diatom species such as *Thalassionema nitzschioides (T. nitzschioides*), *Eucampia zodiacus (E. zodiacus)*, and *Skeletonema costatum (S. costatum)*. The absence of eCA activities in *T. pseudonana* was reported in previous studies using the isotope-disequilibrium [62], Membrane inlet mass spectrometry (MIMS) [16], and potentiometric methods [17,63]. Details of the photophysiological responses in terms of growth, respiration, and photoinhibition of 20 species of marine diatoms to ocean acidification were reviewed by Gao and Campbell [25], outlining further such complexity.

The extent of how much the carbon acquisition strategies of natural phytoplankton assemblages are affected by ongoing ocean acidification has been examined in incubation experiments on board research vessels (Table 1). An early study by Tortell and Morel [64] demonstrated that HCO_3_^−^ uptake in the equatorial Pacific Ocean is regulated by the ambient CO_2_ concentrations, where phytoplankton assemblages did not express eCA under high CO_2_ concentrations (750 µatm). Several studies observed a reduction in eCA activity as a response to high CO_2_ concentrations (800 µatm) in diatom assemblages of the West Antarctic Peninsula [65,66,67] and more recently in the Timor Sea phytoplankton assemblages [18,68]. Contrarily, tolerance of highly variable CO_2_ levels has been observed for diatom assemblages in the subarctic Pacific, indicating that the direct uptake of HCO_3_^−^ dominates carbon uptake for these assemblages [69]. Evidence of direct HCO_3_^−^ uptake has been seen in southern Bering Sea and Ross Sea diatom assemblages, which is estimated to contribute up to 60–95% of total C_i_ uptake [21,39,69], suggesting that the HCO_3_^−^ transport system is probably never completely suppressed under any ocean conditions [21]. In the Southern Ocean, preferred C_i_ sources under elevated CO_2_ are highly variable [70,71,72], whereby phytoplankton assemblages show substantial direct HCO_3_^−^ uptake. Overall, these findings highlight the fact that the effect of future ocean acidification on phytoplankton C_i_ acquisition strategies may vary between oceanic provinces due to the changing composition of phytoplankton assemblages and environmental conditions. An increase in seawater acidity increases the hydration rates by eCA [73]. Meanwhile, future increases in CO_2_ levels would save about 20% of the energy demand for CCMs [74] of diatoms, as less eCA would be required to maintain C_i_ acquisition. Phytoplankton species that possess direct HCO_3_^−^ uptake as their preferred C_i_ may become less CO_2_ sensitive than those relying solely on CO_2_ uptake or indirect HCO_3_^−^ uptake through eCA. More field experiments from different oceanic regions are needed to compile a comprehensive understanding of how marine phytoplankton would acquire C_i_ in the future oceans.

Short-term shifts in phytoplankton species composition with variable CO_2_ concentrations are expected in future oceans. The effect of CO_2_ concentrations on species composition also varies between oceanic regimes. For instance, incubations of equatorial Pacific phytoplankton assemblages resulted in the dominance in diatoms over the prymnesiophyte *Phaeocystis antarctica* under elevated CO_2_ (750 µatm) [56]. Meanwhile, increased CO_2_ levels (800 µatm) would also favour the growth of larger cells (e.g., *Chaetoceros* spp.) over smaller cells (e.g., pennate diatom *Pseudo-nitzschia*) as observed in incubation experiments with Ross Sea phytoplankton communities [75], because larger cells are subject to greater reaction-diffusion limitations [76]. Shifting towards larger diatoms (*Thalassiosira* sp., *T. nitzschioides* and *Nitzschia longissimi*) is also observed in natural phytoplankton assemblages from the Kiel Fjord (Germany) under distinct “greenhouse” conditions (8.5 °C and 990 µatm) [77]. In very different oceanic conditions, combining high CO_2_ and surface solar radiation resulted in declines in the diatom abundance from the South China Sea and their primary productivity [78]. Such taxonomic shifts are likely to be influenced by the physiological mechanisms of C_i_ use by specific species and, therefore, it is essential to fully describe the CCMs of natural phytoplankton assemblages to predict how they will respond to future changes in CO_2_ levels [21]. A shift towards larger cells—as observed for diatoms—could increase the vertical flux of POC and the efficiency of the carbon pump to the deep ocean by forming rapidly sinking aggregates [79]. Moreover, larger diatoms had higher total CA activity for a given Zn- or Cd-limited growth rate, and thus, the cell could be co-limited by Zn, Cd, and CO_2_ at low external CO_2_ concentrations [49]. This is because larger cells have lower cellular Zn due to their lower cell surface to volume ratio, and a greater restriction of a diffusive flux of biologically available dissolved Zn to their surface due to a thicker diffusive boundary layer around their cells [80].

As continuing ocean acidification directly affects the physiology of certain diatoms, it may also indirectly influence their response to other environmental factors including ultraviolet (UV) radiation, light, increasing temperature, or nutrients [25]. The net effect of ocean acidification on marine producers largely depends on the photo-biological conditions (light or UV radiation) [81,82], as well as interaction with rising sea-surface temperatures [83] and probably other variables such as changes in nutrient availability. These environmental factors may have a synergistic or antagonistic effect on the C_i_ acquisition of diatom species. A combination of low light and high CO_2_ reduced the eCA activity of *S. costatum* by 2.5-fold, implying that besides CO_2_, the efficiency of CO_2_ uptake is dependent on the availability of light [84]. This highlights the importance of light in CCMs efficiency.

## 4. Enrichment of eCA in the Sea Surface Microlayer

The hypothetical enrichment of eCA within the sea surface microlayer (SML) was proposed by Berger and Libby [87] in the late 1960s considering the hydrophobic nature of eCA [88]. The SML is a boundary layer between the ocean and the atmosphere, covering a significant fraction of the Earth’s surface [89], and is characterized as a distinct habitat for plankton communities [90]. A high abundance of microorganisms such as picophytoplankton accumulating in the SML compared to underlying water at 1-metre depth has been frequently reported [91,92]. Besides, earlier studies [93,94] have described that the SML is dominated by diatom, cryptophytes, and dinoflagellates species. Indeed, various species of dinoflagellates and diatom are reported to express eCA [18,76,95].

It was suggested that eCA expression in the surface water is associated with surface water ecology [18,96], and so the SML may contain a sufficient amount of extracellular and membrane-bound eCA to enhance the conversion between HCO_3_^−^ and CO_2_ in the boundary layer between the ocean and the atmosphere. Thus, any CO_2_ produced by eCA at the SML would rapidly be utilized by cells and converted to biomass. Berger’s and Libby’s hypothesis remained unanswered for five decades as existing analytical techniques were too insensitive and impractical for immediate shipboard measurements. Using a fluorescent technique [18], we found that the concentrations of eCA in natural seawater are in the nanomolar range (0.10 nM–0.76 nM) and enriched in the SML by a mean of 1.5 ± 0.7 compared to underlying water from 1-metre depth [97]. This finding is supported by Subhas, et al. [50], whereby CA in natural seawater was externally bound and accounted for up to 80% of total CA. Nevertheless, the eCA concentrations observed in Mustaffa, et al. [97] were considerably low based on an estimated value of 1.8–4.8 nM considering that for eCA about 0.3% of its molecular weight consists of Zn [42], and Zn is enriched in the SML by an enrichment factor (EF) of 1.5–4.0 [98]. A short residence time of Zn in the SML [99] and a short lifetime of eCA could explain the low levels in the SML. Meanwhile, a complex enrichment process in the SML [94,100] including wind speed, intense UV radiation, and temperature fluctuation [101], excludes a simple explanation of eCA enrichment and opens up a new research field.

Recently, Watson, et al. [102] pointed out that most computer models underestimate oceanic carbon uptake, partially due to constraints in the measurements of sea-surface temperature. However, using a conservative laminar film model [103], we concluded that the existing nanomolar level of eCA in the SML can enhance CO_2_ exchange by up to 15% [97], which represents 0.37 petagrams (Pg) carbon year^−1^ considering a global estimate of oceanic carbon uptake of 2.5 Pg C yr^−1^ [104]. Based on the EF of eCA per chlorophyll-a from our study [97] and a global concentration of chlorophyll-a (0.1–2.1 mg/m^3^; source: http://oceancolor.gsfc.nasa.gov/, accessed on 5 March 2021) during the cruise, we estimate here that the concentration of eCA in the SML could be in the range of 0.12–1.20 nM (EF = 0.3–3.4) and could contribute up to a 23% enhancement of CO_2_ exchange based on Keller’s model. With this estimation, we suggest that ignoring the enrichment of eCA at the SML further explains why computer models underestimate global carbon uptake rates. However, the enhancement could be less than 23% considering the complexity of the SML and uncertainty in the measurement of air–sea CO_2_ exchange in natural conditions. Further validation is needed as the eCA expression in natural communities is dependent on pCO_2_ conditions, light, and nutrient availability. Besides, the eCA levels may vary between oceanic provinces with different phytoplankton communities and sizes, as diatoms commonly express eCA when demand for CO_2_ outstrips the rate of supply by uncatalyzed bicarbonate to CO_2_ conversion, whereas cyanobacteria do not [38,64].

Life in the SML is challenging as the communities are exposed to intense light, UV radiation, and temperature fluctuations [101], which limit the activity and abundance of photosynthetic organisms [105]. Thus, the efficiency of CO_2_ uptake by phytoplankton in the SML is likely to be affected by UV radiation. In a laboratory experiment, Wu and Gao [85] observed that the eCA activity of *S. costatum* was enhanced by 28% and 24% under UV-A and UV-B radiation, respectively. This was observed at relatively low irradiance (PAR = 161 Wm^−2^) after 1-hour exposure and contributed up to 6% of the photosynthetic carbon fixation rate. However, exposure to higher levels of UV radiation (UV-A + UV-B) for 2 h degraded the eCA by 78%, implying that UV radiation contributes to greater photoinhibition of photosynthesis [85]. Degradation of RubisCO has been observed under similar high UV conditions [106,107]. The light conditions and warming of the ocean (i.e., 200 µmol photons m^−2^ s^−1^ and 25 °C)—predicted future climate conditions [53]—substantially declined the expression of eCA and RubisCO activity in *T. weisflogii* but not in *P. tricornutum* [86]. This suggests that climate-related feedbacks are species-specific and *T. weisflogii* may have benefits in terms of its growth in the future ocean.

With future changes of UV flux to the ocean [108] as well as an increase in sea-surface temperature, it is reasonable to expect a lower eCA expression in SML communities with a consequence of decreased CO_2_ uptake by the ocean and thus decreased air-sea CO_2_ exchange. Despite the relevance of the SML in air–sea CO_2_ exchange [109,110] and the fact that phytoplankton at the near-surface layer have been suggested to control the air-sea CO_2_ equilibrium [111], the sensitivity of the SML communities to ocean acidification and combination effects including UV radiation and temperature are still largely unexplored [112]. Because of the unique location of the SML between the ocean and atmosphere, the communities in this layer are likely to be the first to be exposed to climate-related changes. For instance, previous studies have shown that light limitation affects growth rates and biomass in SML communities [95], and high nutrient loads changed the density and composition of SML communities [113]. Incomplete understanding of the C_i_ acquisition strategy in the SML community’s response to future climate change leads to difficultly in predicting the global chemical enhancement of CO_2_ and biogeochemical cycling by eCA. Overall, futures investigations are necessary to get a mechanistic understanding of phytoplankton and its carbon acquisition strategies in the dynamic SML and upper ocean layer.

## 5. Conclusions

Our review highlighted the current knowledge and gaps in the knowledge about the role of eCA in the changing ocean. eCA activity and concentrations are affected by environmental stressors such as ocean acidification and UV radiation as well as changing light conditions. Thus, eCA potentially serves as a biochemical indicator in biomonitoring programmes and could be used for future response prediction studies in changing oceans. As most of the studies were carried out in the short term (i.e., days), we propose that studies aiming for a long-term response of diatoms to environmental changes should be conducted in the future. We also suggest including the near-surface layer (including the SML) communities in a research effort to study physiological responses towards ocean acidification, UV radiation, temperature fluctuations, as well as nutrient limitations. Such studies will provide further insights into the global chemical enhancement of CO_2_ and biogeochemical cycling in future oceans. Furthermore, advancing technology such as analytical methods, molecular tools, and bioinformatics are needed to resolve the metabolic roles of eCA in photosynthetic organisms. The application of such tools will be crucial to widening the perspective of eCA studies in natural seawater and predicting changes for the future oceans, including the interaction of multiple concurrent changes such as pH and light conditions. In this context, the SML—covering 71% of the Earth’s surface—seems to be a good candidate with more drastic changes likely to occur. Overall, eCA from different oceanic provinces remains to be explored to further improve computer models of marine carbon cycling, including the oceanic CO_2_ uptake as well as their response in a future ocean.

## Figures and Tables

**Table 1:** Studies on the effect of ocean acidification, UV radiation, light, warming, and combination effect on carbon acquisition strategies of phytoplankton through laboratory (diatom species) and incubation experiments on board research vessels (natural phytoplankton assemblages).

Experiment	Phytoplankton	Results	Observation Types	References
Ocean Acidification	*Thalassiosira weissflogii*	Decline in eCA expression with increasing CO_2_ concentrations	Laboratory	[26,27,28]
	*Phaeodactylum tricornutum*	Decline in eCA expression with increasing CO_2_ concentrations	Laboratory	[26,27,28]
	*Thalassiosira pseudonana*	eCA activities were not affected by CO_2_ concentrations	Laboratory	[16,17,40,62,63]
	*Thalassiosira nitzschioides*, *Eucampia zodiacus*, and *Skeletonema costatum*	eCA is important in bloom-forming diatom species. eCAdecline with increasing CO_2_ supply (800 µatm)	Laboratory	[40]
	Antarctic phytoplankton *(Chaetoceros debilis, Pseudo-nitzschia, Fragilariopsis kerguelensis*, and *Phaeocystis antarctica)*	Preferences for C_i_ sources are partly species-specific. eCA activities of *Pseudo-nitzschia and P. antartica* increased under low pCO_2_ but the eCA activities of *C. debilis* and *F. kerguelensis* were unaffected by pCO_2_	Field experiment	[70]
	Equatorial Pacific Ocean natural assemblages	No eCA expression under high CO_2_ concentrations (750 µatm)	Field experiment	[64]
	West Antarctic Peninsula diatom assemblages	Decline in eCA expression with increasing CO_2_ concentrations (800 µatm)	Field experiment	[65,66,67]
	Timor Sea phytoplankton assemblages	eCA decreased faster in the low pH/high CO_2_ treatment compared to the in situ CO_2_ treatment	Field experiment	[18,68]
	Subarctic Pacific diatom assemblages	eCA activity does not respond to increasing CO_2_, indicating direct HCO_3_^−^ uptake	Field experiment	[69]
	Southern Bering Sea	eCA activity does not respond to increasing CO_2_, indicating direct HCO_3_^−^ uptake	Field experiment	[21]
	Ross Sea diatom assemblages	Regulation of C_i_ uptake by phytoplankton is dependent on seasonal bloom	Field experiment	[69,75]
	Southern Ocean phytoplankton assemblages	Substantial direct HCO_3_^−^ uptake by phytoplankton	Field experiment	[71,72]
UV radiation	*Skeletonema costatum*	Degradation of eCA by 78% after 2 h exposure	Laboratory	[85]
Light + Ocean acidification	*Skeletonema costatum*	Higher eCA activity under low CO_2_ and high light. Efficiency of CO_2_ uptake by *S.costatum* is dependent on the availability of light in addition to CO_2_	Laboratory	[84]
Light + warming	*Thalassiosira weissflogii* and *Phaeodactylum tricornutum*	Declined in eCA expression in *T. weisfligii* but not *in* *P. tricornutum*	Laboratory	[86]

## Data Availability

Not applicable.
